# Changing insect populations: New insects, old venoms

**DOI:** 10.1016/j.jacig.2025.100559

**Published:** 2025-08-21

**Authors:** David B.K. Golden

**Affiliations:** Johns Hopkins University School of Medicine, Baltimore, Md

**Keywords:** Insect sting, anaphylaxis, Hymenoptera, venom allergy, hornets, climate change

For 45 years, the insect extracts that are commercially available in the United States for diagnosis and treatment of insect sting allergy have remained unchanged. When they were introduced, honey bee (HB), yellowjacket (YJ), hornet, and wasp venom extracts were expected to cover all the common causes of sting anaphylaxis when used for diagnostic testing or venom immunotherapy (VIT) treatment. For imported fire ants (IFAs), the long-available whole body extracts were believed to provide sufficient allergenic activity for diagnosis and treatment, unlike the whole body extracts of the winged Hymenoptera, which were demonstrated to be no better than placebo. Over the years, however, there have been exceptions reminding us that in addition to the species represented in the available extracts, there are myriad other species that can cause allergic reactions—and that known species can migrate to new areas. The latest such threat comes from an Asian hornet, as described by Giangrande et al in this issue of the *Journal of Allergy and Clinical Immunology: Global*.^1^

Invasive species and climate change continue to alter the landscape of insect sting allergy throughout the world and in the United States. More than 100 years ago, IFAs (*Solenopsis* spp) invaded the United States, and they have since become one of the leading causes of insect sting anaphylaxis. In the past decade, they have also invaded China and Australia and have started to appear in Europe as well, bringing new problems to regions that were not prepared to manage them.^2^ Even in their existing habitat, increasing average temperatures will allow them to move farther north than ever before. On a positive note, the increasing need for IFA extracts in new markets will perhaps lead to development of a *Solenopsis* venom extract, similar to what has occurred with the development of jack jumper ant (JJA) (*Myrmecia pilosula*) venom for immunotherapy in Australia.

Other insect species have also invaded the United States in the past. Africanized HBs (“killer bees”) entered the United States in 1990 despite enormous efforts to stop their migration through Central America. Although their venom is no different from HB venom, they present increased danger to those with allergies because of their propensity to swarm and sting in large numbers.

*Polistes dominulus*, the Mediterranean wasp, invaded the United States about 45 years ago and has been expanding its territory and numbers over the years. It has become a common source of problems, as it dominates the native species in some areas and is easily and often confused with *Vespula* (YJs) because of its bright yellow and black markings. Notably, the venom of *P dominulus* shows some unique allergenic activity such that some patients could have specific sensitization that does not cross-react with other *Polistes* species and could therefore be missed on diagnostic testing with commercial *Polistes* venom extract (and could thus escape protection with *Polistes* VIT).^3^

The importance of other less common species came to light very soon after the introduction of Hymenoptera venom extracts. In 1981, just 18 months after venom extracts had been approved, the US Food and Drug Administration agreed to make 1 addition to the species that are included in the YJ venom mix. The commercial YJ venom extract is actually a mix of 4 to 6 species that represent the most common *Vespula* species in the United States and are expected to cover a wide range of other YJ species.^4^ However, *V squamosa* was known to be more prevalent in Florida and was shown to have some unique allergenic activity. To ensure optimal protection of patients in that region, *V squamosa* was specified as a species required to be included in the YJ venom mix, and it has been included in the mix since 1981. *Polistes* venom extract is also a mixture of 3 to 5 species, not including *P dominulus*. However, there are other *Polistes* and related wasp species that may differ antigenically from those included in the commercial extract. Another example of the effects of climate change on insect populations is the observation that frequent flooding of the Mississippi River Delta around New Orleans has virtually eliminated *Vespula* species (many of which nest underground), leaving *Polistes* wasps as the dominant Hymenoptera species in the area, including species that are not used in the commercial venom extract.^5^ In contrast, climate change in Alaska may be related to observations of YJs having longer seasons and increasing numbers.^6^

Asian hornets have also become increasingly invasive as a result of migration and/or accidental introduction. Asian giant hornets [*Vespa mandarinia*] appeared in northwestern United States in 2019 but are said to have been eradicated there. Another Asian hornet (*Vespa velutina nigrithorax* [VVN]) has expanded its territory into northwest Asia, and in recent years, it has become an increasingly common culprit causing insect sting anaphylaxis in Western Europe, as well as a scourge of the HBs on which they prey.^7^ They are reported to have arrived in western France in shipping boxes from China sometime around 2004, and they spread through France and Spain over the subsequent 10 years before arriving in Germany and the United Kingdom sometime around 2015. In the United States, there is evidence that VVN hornets established nests in Georgia and South Carolina in 2024, and there is concern that climate change may accelerate their migration throughout the United State and northern Europe.^8^ The report by Giangrande et al in this issue of the *Journal of Allergy and Clinical Immunology: Global* reminds us of the aggressive nature of these hornets and the threat the they pose to patients with insect allergy.^1^

The problem is not only one of concern for accurate diagnosis of patients with insect sting allergy; it also has important implications for treatment. There are common expectations, sometimes misplaced, that VIT with commercial venoms will provide significant protection against allergic reactions to all insects of related species. For example, because many patients with bumble bee sting allergy also showed a positive reaction to HB venom, it was assumed that VIT with HB venom would protect them against anaphylaxis in response to a bumble bee sting. Studies of greenhouse workers proved this to be untrue.^9^ The report by Giangrande et al exposes a similar problem with hornet allergy.^1^ There have been no commercial extracts of true hornet venoms (genus *Vespa*) because historically, the common European (*V crabro*) and Asian (*V orientalis*) hornet venoms showed almost complete cross-reactivity with *Vespula* venoms, and VIT with *Vespula* venoms is considered adequate to protect against reactions to these stings. There have been previous reports suggesting that VIT with *Vespula* venoms would also protect against anaphylaxis in response to VVN stings.^10^ The report by Giangrande et al^1^ demonstrates that this is not always the case, and it warns us all to beware of expectations of cross-species protection.

We need more commercial venom extracts to represent the range of species causing sting reactions in the field. There continue to be patients with a clear history of sting anaphylaxis but negative results of tests for venom allergy and mast cell disorders. The frequency of anaphylaxis in response to insect species other than those included in commercial extracts is unknown, but the increasing number of species being identified as potential culprits points to a need for a greater variety of venom extracts. The impact on patient care is significant: we are not only missing the diagnosis in some patients but are also failing to protect others for lack of specific venoms. There are many different species of oak, grass, or birch pollen extracts available commercially in the United States despite their extensive cross-reactivity, yet we are limited to a small number of venoms that were selected in the 1970s. As suggested by Carlson and Fox, allergists should collaborate more closely with entomologists to identify potentially relevant species in different areas and emulate our colleagues in Italy, Australia, and Spain who moved quickly to develop and license specific new venom extracts for “new” insect species (*P dominulus*, JJA, and VVN, respectively).^5^ This will be a challenge to entomologists, allergists, and US Food and Drug Administration regulatory committee members, who must collaborate to serve the current and future needs of our patients.

## Disclosure statement

Disclosure of potential conflict of interest: D. Golden receives consulting fees from 10.13039/100011033Thermo Fisher and royalties from UpToDate.
